# Correction: When revealed after the fact, selfish intentions undermine prosocial actions in 5-year-olds

**DOI:** 10.1371/journal.pone.0348092

**Published:** 2026-04-27

**Authors:** Kayley Dotson, Michael Tomasello

The first author, Kayley Dotson, is incorrectly noted as the corresponding author. The correct corresponding author is Michael Tomasello. Michael Tomasello’s email address is: michael.tomasello@duke.edu.
