# Supplementary material for: When revealed after the fact, selfish intentions undermine prosocial actions in 5-year-olds
Source: PLoS One. 2026 Mar 18;21(3):e0344731. doi: 10.1371/journal.pone.0344731 (PMC12998847; doi:10.1371/journal.pone.0344731)
Supplement: S2 File — Exact script used for all sessions. (DOCX) [file pone.0344731.s002.docx]

E1 warms up with child. Introduces Alex (elephant) with boring warm up question.

E1: “Alright, child, let’s go to a different room, and I can show you how to play a game. Does that sound fun?”

E1: “Alright, here we go!”

*begin video*

E1: “Okay, we’re here! Let me show you how to play!”

E1: “Oh, wait, I think I hear someone!”

*P1 enters*: “Hi! My name is Sam! I came to play today, too! Is that okay?”

E1: “Hi, Sam! Sure! Thanks for coming! We have a friend here to play today, too!”

P1: “Oh, hi! It’s nice to meet you!”

E1: “Today, we’re going to play daxing!” *pull out buckets*

E1: “Let me show you how to play!”

E1: “Okay! There’s a ball, and we can drop it in the green bucket or the black bucket. If we drop it in the green bucket, it makes this sound! If we drop it in the black bucket, it makes this sound! That’s how you play! Now it’s time to play daxing!”

E1: “Hmm… Sam’s going to decide who gets to play. It’s his choice. You tell me who’s going to play Sam?”

P1: “Oh daxing looks like fun! I want to play, but maybe I should let you try. Hmm… Okay, you’re my friend so I’m going to let you try daxing. My new friend can play.”

E1: “Alright, do you want to try?”

E1: “Okay, you go ahead and try it! Alright, let’s try the black bucket first! I’ll say 3, 2, 1, and then you say go, okay? 3, 2, 1… Lets try the green bucket” *play several rounds* *puppet gets smaller and goes to corner – definitely not playing* DROP

*After several rounds, E1 pauses*

P1: *excitedly* “The game’s over now, right?”

Pan over to side of the room where puppet is now standing to show plate with cookie (empty plate for neutral)

E1: “Yes, we’re done playing daxing!

P1: “Okay! I hope you had fun playing daxing…

| Undermine | Neutral |
| --- | --- |
| P1: I only let you play because I wanted to get a cookie instead! I get a cookie! This is so much better than daxing. Yay! | P1: I only let you play because I wanted you to have fun! You had fun! I’m so glad I let you play daxing. Yay! |

P1: “Oh, I have to go now! Bye”

E1: “Okay, bye! Alright, let’s go back into the other room now!”

E1: *Back in room* “Alright! I liked daxing. What did you think?”

DVs: Liking, forced choice, give resource or keep?, commitment

Commitment:

“I think Sam might come back soon. You remember him, right?

Okay, great.

I have a game for us to play. We can go ahead and play the game with just you & me, or we could wait for Sam so that we all can play the game.

Do you want to wait for him?”

*Wait 10 seconds*

“Hmm, I wonder where he is. Do you want to keep waiting for Sam?”

*Wait 10 seconds*

“I’m not sure where he is. Do you want to keep waiting for him?”

*Wait 10 seconds*

“I wonder where Sam is… Do you think we should keep waiting for Sam? ”

*Wait 10 seconds*

“Hmm I don’t know if Sam is coming back. Do you think we should wait or go play the game?

*Wait 10 seconds*

“I don’t think Sam is coming back. Do you want to keep waiting for Sam or go play the game?”

*Wait 10 seconds*

“It looks like Sam isn’t coming back. I guess Sam was busy doing something else. Let’s go play the game!”
