# Supplementary material for: When revealed after the fact, selfish intentions undermine prosocial actions in 5-year-olds
Source: PLoS One. 2026 Mar 18;21(3):e0344731. doi: 10.1371/journal.pone.0344731 (PMC12998847; doi:10.1371/journal.pone.0344731)
Supplement: Attachment — Submitted filename: IPA_ResponseToReviewers.docx [file pone.0344731.s004.docx]

Title: When revealed after the fact, selfish intentions undermine prosocial actions in 5-year-olds

*PLOS ONE*

Dear Dr. Asami Shinohara,

Thank you for the opportunity to submit a revision of our manuscript entitled “When revealed after the fact, selfish intentions undermine prosocial actions in five-year-olds”. We appreciate the thoughtful and constructive feedback that you and the reviewers provided. We have revised the manuscript to properly match PLOS ONE’s style requirements, moved our ethics statement to the proper location, removed all identifiers from our data, and added captions for Supporting Information. We have worked to address each comment and believe the manuscript is far stronger now. Below, we provide a point-by-point response for each reviewer comment.

Reviewer 1

1. On page 5, line 84, the authors state that they are interested in what happens when children initially believe one thing about a partner’s intentions and then receive contradictory evidence. They also mention they aim to investigate whether children engage in moral belief revision. However, the methodology does not include a measure of children’s beliefs about the partner’s initial intentions, nor does it directly assess belief revision. It is therefore unclear whether children inferred any intention at all prior to its revelation, or whether they had already assumed a selfish motive from the beginning.

**Response:** Thank you for your thoughtful feedback. We agree that our original framing overstated what our design measured and have now removed any claims that our study explores belief revision. Now, our only mention of belief revision comes in the discussion as a point for future research:

“Another explanation for why reciprocity, but not social evaluation or social bonding, was affected may lie in evidence from belief revision. It is possible that children revised their interpretation of the prosocial act itself, viewing it as less genuine, but without updating their belief about the agent themselves. Preschool children revise their beliefs when considering counterevidence in their physical and social worlds (Kimura & Gopnik, 2019; Migosa et al., 2020). While our design did not directly measure belief revision, the pattern is consistent with children updating their moral beliefs regarding the prosocial action. Future work should test these processes explicitly to understand how children may revise their beliefs in the moral domain.”

2. I suggest the authors provide more justification for focusing solely on 5-year-olds, rather than including a broader age range. How might developmental differences affect the findings?

**Response:** Thank you for raising this point. We now explicitly justify our focus on 5-year-olds in the introduction. We also added a section in the discussion to mention the need for future work to explore a larger age range. In the introduction we have added the following justification:

“Exploring how 5-year-olds reason about prosocial motivations may be of particular interest due to recent theoretical accounts and empirical findings. Recent theoretical models propose two major shifts in prosocial development: from early intrinsic motivation to strategic concerns by age five (Engelmann & Rapp, 2018; Leimgruber, 2018; Robbins & Rochat, 2011; Warneken et al., 2019) and then from strategic concerns to internalized prosocial and fairness norms around and beyond age five (Blake et al., 2015; House, 2018; Hepach and Tomasello, 2025). Also, children’s prosocial behaviors at this age may be more stable across culture. Across six societies, prosocial behaviors did not change as a function of culture until around age six (House et al., 2013). Five-year-old children therefore are at a transitional point, wherein they can understand and strategically engage in prosocial behaviors and reciprocity but are not yet necessarily fully guided by moral or cultural norms. This age therefore provides an ideal window into investigating how intention information shapes prosocial behavior and reciprocity.”

In the discussion, we have added possible developmental differences and the need to explore these age ranges further: “One limitation of the study is that the sample size was small and only included one age group: 5-year-olds. Still, our effect size was moderate-to-large even given the small sample, but future research with larger samples should be conducted to replicate the findings presented here. Future research may also include a wider age range. Given that children younger than five often focus on outcomes instead of intentions and that theory of mind reasoning is still developing through age four (Astington & Jenkins, 1995; Baird & Astington, 2004; Wellman et al., 1990), we would expect that 3- to 4-year-olds would show a weaker or inconsistent sensitivity to E2’s selfish intention. On the other hand, we would expect older children to rely more on intentions and norm reasoning (Killen & Smetana, 2013) that would lead to a similar, though potentially stronger sensitivity to E2’s selfish intention.”

3. How was the sample size determined? Is it possible that the study is underpowered?

**Response:** We have included the following justification for our sample size in our methods section: “A sample size of 48 was selected as it is conventional in the field and, in this case, slightly larger given that the study focused on a single age group (see Beeler-Duden & Vaish, 2020; Vasil et al., 2024).”

4. I found the procedure somewhat difficult to follow. In particular, the role of E3 was not immediately clear. I recommend providing a clearer description of E3’s function in the procedure.

**Response:** Thank you for pointing this out. We have added the following additional information about E3’s role in the procedure: “E3 was introduced at the beginning to serve as a neutral, somewhat positive agent. E3 would later be included as a potential play partner in our dependent measures.”

5. The prosocial act performed by E2—allowing the child to play first—is relatively subtle. Would the results differ if the prosocial behavior were more direct or explicit, such as sharing or helping?

**Response:** We agree that the prosocial manipulation is rather subtle. We have added a paragraph in our discussion to expand on this idea and highlight the need for future research on this:

“The prosocial behavior performed by E2, letting the child have a turn in the novel game, was relatively subtle. This subtle prosocial behavior is less overt than other more robust prosocial behaviors, like sharing or helping. It is therefore impressive that even this low-cost act was undermined once a selfish intention was revealed. More explicit prosocial behaviors may create increased feelings of obligation or expectations of reciprocity, potentially producing even larger reciprocity effects than those observed here. However, future research could explore the differences in varying prosocial acts.”

6. In the results section, I suggest including descriptive statistics for the liking, forced-choice, and social bonding measures. Additionally, it would be helpful to conduct analyses comparing performance to chance for each condition. For example, did children reciprocate significantly above chance in both the neutral and undermining conditions?

**Response:** Thank you for suggesting this. We have now added a table of descriptive statistics for all DVs, included these descriptives throughout our results section, and conducted and included analyses comparing reciprocity to chance.

Reviewer 2

Major Concerns
1. Research Framing
The introduction and discussion appear to be structured around two key concepts: reciprocity and belief revision. While I can understand that this is a study about reciprocity, the study's procedures and results do not support the claim that this is a study of belief revision.
The study's procedures only measure children's behavior and evaluations of/choices about the agent after intention disclosure. Therefore, the study fails to establish: (1) what beliefs children initially held about the prosocial agent, and (2) whether these beliefs changed "before and after" the presentation of intention information. The relationship between beliefs about prosocial behavior and evaluations of the agent (or whether such evaluations are included in beliefs) also remains unclear.
Therefore, what can be concluded from this study's results is simply that children's reciprocal behavior decreases when selfish intentions behind prosocial behavior are revealed. Consequently, the introduction and discussion need to be restructured to focus specifically on children's reciprocity and the relationship between prosocial behavior and intention.

**Response:** Thank you for pointing this out. We have now removed any claims that our study explores belief revision. We also have reframed the study to highlight prosocial preferences, reciprocity, and intentions and have arranged our introduction accordingly. Now, our only mention of belief revision comes in the discussion as a point for future research:

“Another explanation for why reciprocity, but not social evaluation or social bonding, was affected may lie in evidence from belief revision. It is possible that children revised their interpretation of the prosocial act itself, viewing it as less genuine, but without updating their belief about the agent themselves. Preschool children revise their beliefs when considering counterevidence in their physical and social worlds (Kimura & Gopnik, 2019; Migosa et al., 2020). While our design did not directly measure belief revision, the pattern is consistent with children updating their moral beliefs regarding the prosocial action. Future work should test these processes explicitly to understand how children may revise their beliefs in the moral domain.”

2. Critical Errors in Statistical Analysis
The reporting of analytical results in this paper contains several critical errors and insufficiencies.
Insufficient Data Reporting: For the Liking score, Forced Choice, and Social Bonding results, only analytical results (mainly p-values) are described, making it completely unclear what the actual data patterns were. For example, regarding the Liking score, while we understand there was no group difference, it's unclear whether this means the agent was liked in both groups (mean scores close to 7) or disliked (close to 1). Without these detailed results, readers struggle to understand the findings, and any discussion derived from the data becomes thin or erroneous.

**Response:** Thank you for suggesting this. We have now added a table of descriptive statistics for all DVs, included these descriptives throughout our results section, and conducted and included analyses comparing reciprocity to chance.

Social Bonding Analysis Errors: More problematic is the analysis and interpretation of Social Bonding results:
1. Incorrect test statistic: The authors report "t = -0.814" for ordinal logistic regression, but ordinal logistic regression uses Wald z-statistics or likelihood ratio tests, never t-statistics. The correct analysis and statistical values need verification.

**Response:** Thank you for highlighting this error. We have fixed this in the results section to show the accurate Wald z-statistic.

2. Inappropriate interpretation of non-significant results: Despite the model being statistically non-significant (evident from both the p-value and 95% CI crossing zero), the authors inappropriately interpret odds ratios. When results are non-significant, such effect size interpretations should be avoided.

**Response:** Thank you for highlighting this. We have removed any interpretations of the non-significant results.

3. Sign inconsistency: The authors state log odds = -0.42, but then write "exp(.42) = 1.52 times higher," with the sign reversed. Following the log odds value, it should be "1.52 times lower."

**Response:** We have removed this entirely, so as not to make any erroneous claims.

4. Misinterpretation of ordinal logistic regression: The odds scale interpretation treats this as a binary comparison ("not waiting at all vs waiting after one or more prompts"). However, the coefficient represents the change in cumulative log odds for being in lower versus higher categories across all possible thresholds, not just a single binary comparison.

**Response:** Again, we have removed this entirely, so as not to make any erroneous claims.

3. Insufficient Discussion
The discussion is superficially shallow overall, with little reference to prior research, resulting in mostly simple result descriptions and mere speculation. This is particularly problematic given that belief revision cannot be addressed. For example, regarding why group differences were observed in reciprocal behavior but not in liking or social bonding, many points remain to be discussed: why such results occurred, whether both results would be expected to align in older children or adults, and if so, what developmental factors might be involved. Even Vaish et al. (2018), cited in this paper, found that behavioral and evaluative results did not necessarily align, but they provided sufficient discussion of this point. Without deepening the discussion by referencing such prior research, this paper remains merely a report of results, with limited scholarly contribution.

**Response:** We appreciate your feedback on our discussion and have strengthened this section by adding a few new paragraphs and several citations to support our ideas. We now include authors like Blake, Shaw, Killen, Astington, Baird, Wellman, and House among others to discuss alternative explanations, potential age-related differences, and theoretical positioning.

Other Concerns & Minor comments
Unclear sample size determination:
While the study included 48 participants, the rationale for determining this sample size is not provided. Although 48 participants may not be considered too small, demonstrating that the sample size and analyses are appropriate is essential for establishing this as rigorous scientific psychological research.

**Response:** We have included the following justification for our sample size in our methods section: “A sample size of 48 was selected as it is conventional in the field and, in this case, slightly larger given that the study focused on a single age group (see Beeler-Duden & Vaish, 2020; Vasil et al., 2024).”

Disorganized literature review:
Throughout the introduction, the positioning of prior research findings and this study is poorly organized, giving the impression of a list of prior studies related to keywords like prosocial behavior, moral judgment, intention, and reciprocity. What's important for deriving this study's research question is: (1) children engage in prosocial behavior and evaluate prosocial people positively, (2) reciprocity toward others' prosocial behavior is observed in children, and (3) intention information is used in evaluations of others and reciprocity. The literature review should focus on these points, then explain how this study's questions differ from prior research.

**Response:** Thank you again for your thoughtful reading of the introduction. We have reorganized the introduction to largely follow the suggestions you laid out. We introduce literature on prosocial behaviors, reciprocity, and intentions and frame our study with these literatures.

Unclear emphasis on "after":
Several places emphasize "after" in "selfish intention revealed after a prosocial behavior" with italics, but it's unclear why "after" is considered so important. This study did not examine the effects of when selfish intentions are revealed, nor was this timing emphasized in discussions of prior research. While this study does have a structure where selfish intentions are revealed after prosocial behavior, emphasizing "after" may mislead readers about the main research objective (i.e., whether selfish intent affects reciprocity).

**Response:** As mentioned above, we have now removed any claims that our study explores belief revision. We also have reframed the study to highlight prosocial preferences, reciprocity, and intentions and have arranged our introduction accordingly.

Missing exclusion criteria:
The raw data suggests that several participants were excluded from analysis, but there is no description of exclusion criteria or the number of excluded participants. If specific exclusion criteria were established, they need to be clearly stated.

**Response:** Thank you for pointing this out. We have added the following exclusions in our methods: “In addition to the 48 children in the final sample, nine participants were excluded due to distraction during the manipulation (4), strong preference for one puppet’s name (4), experimenter error (2), or failure to complete the task (1).”

Reviewer 3

1) The “neutral” condition is not neutral. The control puppet states, “I only let you play because I wanted you to have fun,” which is explicitly other‑regarding and morally positive, not neutral. This makes the contrast “self‑regarding vs. other‑regarding,” not “undermining vs. neutral,” and it may inflate differences in reciprocity by juxtaposing a plainly benevolent motive against a plainly selfish one. I recommend renaming conditions accordingly and, ideally, adding a true neutral motive.

**Response:** Thank you for highlighting this. We agree with you that “Neutral” may not have been the best choice for the name of our condition. We have thus renamed the condition to be “Prosocial.” We have changed this throughout the entirety of the manuscript to now contrast an “Undermining condition” versus a “Prosocial condition.”

2) Alternative explanation: equity repair rather than “moral belief revision.” When E2 announces they got a cookie for letting the child play, children may infer an advantage accrued to E2. Choosing not to give the additional toy to E2 can be read as equity restoration (a fairness decision), not necessarily a revision of moral beliefs about intentions per se. The present design cannot distinguish “motive‑based discounting” from “resource‑balancing.”

**Response:** We appreciate you bringing up this point and have included a paragraph in our discussion dedicated to this alternative explanation:

“An alternative explanation for our findings is that children may have been sensitive to equity restoration and not intention information alone. When E2 revealed that they had received a cookie for letting the child play, children may have inferred that E2 already received a benefit and did not require further reciprocation. Not reciprocating, then, may be viewed as an attempt to make distributions fair or equitable, which children are highly attuned to from a young age, especially when a distribution is unfavorable to them (Blake & McAuliffe; Shaw & Olson, 2012). Further research should be conducted to further parse apart these explanations.”

3) Primary effect size and its reporting. The paper reports p = .009, but no effect size. Please report also an effect size.

**Response:** This is a great point. While the Fisher exact test did not initially allow us to properly estimate an odds ratio because of a zero cell, we completed a Spearman correlation to estimate effect size, which we report in our results. We also explain this process and the rationale for it.

“Because no participant chose ‘Keep’ (no reciprocity) in the Prosocial condition, resulting in a zero cell, the odds ratio could not be reliably estimated. To obtain an interpretable effect size, we conducted a Spearman’s rank correlation between condition (0 = Prosocial, 1 = Undermine) and Sharing (0 = No reciprocity, 1 = Reciprocity) to estimate effect size. This test revealed a significant association and a medium-to-large effect size (ρ = -0.41, *p* = 0.004).”

4) Statistical inconsistency in the social‑bonding analysis. In the Results you write that the ordinal‑logit coefficient for the undermining condition is −0.42 (95% CI [−1.45, 0.59]) but then interpret the odds as exp(.42) = 1.52 times higher—this reverses the sign of the coefficient and converts a non‑significant, negative effect into a positive one. I think the correct transformation of −0.42 is exp(−0.42) ≈ 0.66 (i.e., ~34% lower odds), and the effect is non‑significant and should be interpreted accordingly.

**Response:** We have removed any interpretations of the non-significant results, so as not to make any erroneous claims.

5) Methodological details. You report a final N = 48 (24/condition) but do not state how many children were initially recruited, how many were excluded (e.g., those who did not say “now”), or why. CONSORT‑style accounting (or a simple flow diagram) is needed to assess selection bias. Please also specify how condition was randomly assigned, whether assignment was balanced across time of day/experimenter, and whether the live experimenter (E1) was blind to condition during outcome collection. Pre‑registration (if any) should be declared; otherwise, please say so explicitly.

**Response:** We have added the following exclusions in our methods: “In addition to the 48 children in the final sample, nine participants were excluded due to distraction during the manipulation (4), strong preference for one puppet’s name (4), experimenter error (2), or failure to complete the task (1).”

We have also added information about condition assignment, blindness to condition, assignment to experimenter, and pre-registration in our methods section.

“Participants were randomly assigned to a condition prior to beginning the study. E1 was not blinded to condition, as she needed to know the condition in order to run the proper manipulation.”

“E1 was the same experimenter for all sessions.”

“The study was approved by the University Institutional Review Board. This study was not pre-registered.”

6) Theoretical positioning. There is quite a lot of literature on moral preferences among adults, which I think is relevant here (e.g., Alger & Weibull, 2013; Capraro & Rand, 2018; Capraro & Perc, 2021; Basic & Verrina, 2024).

**Response:** Thank you for providing these citations. They were greatly helpful in our theoretical positioning, and we have incorporated them into a new paragraph in the introduction to compare with strategic reciprocity and norm concerns in children:

“Research from behavioral economics may point to similar strategic processes working in in adults. Adults show preferences for prosociality and morality in their economic decisions, though this reasoning may conflict with one’s personal gain. Beyond social norms, adults may also reason with personal norms driven by an individual’s standard for what is right or moral (Bašić & Verrina, 2024; Capraro & Rand, 2018). Adults then reason with both selfishness and a preference for moral behavior (Capraro & Perc, 2021), a balance represented by the theoretical evolutionary model known as the homo moralis (Alger & Weibull, 2013). Together, these findings in adults demonstrate the importance of evaluating both prosocial behaviors and intentions. Understanding the development of these processes working together in childhood is a crucial and unexplored question.”

Reviewer 4

My biggest concern is the review of the literature, both in breadth and recency. The reference list is extremely brief for such a well-studied topic, with one-fourth coming from the same research group and no citations beyond the year 2020. Several arguments made seem to be lacking appropriate citations. For example, the sections on children’s sensitivity to intentions can include works by Baird, Astington, Killen, Carey, Nielsen, Blake, and many more. Research by Baillargeon, Smetana, Harris, and others come to mind on the topic of the early development of reciprocity. The authors might also consider discussing connections to moral reasoning frameworks (e.g., Killen, Rutland). The literature included is also heavily US-centric, when many of the findings discussed have been replicated in other countries, which significantly undermines the impact of the work. On the topic of contradictory evidence, work on children’s ability to consider counterfactuals may help inform hypotheses and/or make sense of the results. Overall, the research groups I’ve suggested is only a small portion of the relevant literature.

**Response:** Thank you for your comments. We have completely reorganized and rewritten both our introduction and discussions in order to incorporate more, newer, and cross-cultural research to back up our claims. We have included citations from all authors suggested and many more. We think that our paper has greatly benefitted from this revising, so thank you for all of the suggestions.

The methodology and results section were very brief and skewed informal in tone. Despite a very small sample size, there is no discussion of power analysis to support or justify it; as a result, I am skeptical about the rigorousness of the statistical analyses conducted. There is no discussion of preregistration -- and if that is the case this should be acknowledged in the main text. Statistical reporting do not follow APA style. Description of the measures are inconsistent (for example, for social evaluation the authors specify it is forced choice, but do not elaborate for the reciprocity measure).

**Response:** We have now provided a justification for our sample size and have acknowledged that this study was not pre-registered. We have bolstered our results section with more statistical analyses and descriptive statistics in order to tell a more complete story of our results. We have also described measures in more detail and clarified portions of our procedure for consistency.

For discussion, the exploration of why they find a significant difference in reciprocity but not social evaluation is very brief and could be expanded – likely after a more thorough literature review is conducted on the topic. There is also no explicit discussion for the social bonding results. Overall, the contribution is difficult to judge due to the lack of depth in interpreting the results.

**Response:** We thank you for your feedback on our discussion and have strengthened this section by adding a few new paragraphs and several citations to support our ideas. We now include authors like Blake, Shaw, Killen, Astington, Baird, Wellman, and House among others to discuss alternative explanations, potential age-related differences, and theoretical positioning.

Thank you all again for the careful attention the editor and reviewers gave our manuscript. We believe that our manuscript is improved and stronger thanks to the feedback we received.

Sincerely,

The Authors
