# Supplementary material for: When revealed after the fact, selfish intentions undermine prosocial actions in 5-year-olds
Source: PLoS One. 2026 Mar 18;21(3):e0344731. doi: 10.1371/journal.pone.0344731 (PMC12998847; doi:10.1371/journal.pone.0344731)
Supplement: Attachment — Submitted filename: IPA_ResponseToReviewers_2.docx [file pone.0344731.s005.docx]

Title: When revealed after the fact, selfish intentions undermine prosocial actions in 5-year-olds

*PLOS ONE*

Dear Dr. Asami Shinohara,

Thank you for the opportunity to submit revisions for our manuscript entitled “When revealed after the fact, selfish intentions undermine prosocial actions in five-year-olds”. We appreciate the careful feedback provided to us for this round of revisions. Reviewers 1 and 3 were satisfied with our revisions, but Reviewer 2 still had some concerns. We have addressed each comment provided by this reviewer. Below, we provide a point-by-point response for each comment.

Reviewer 2

1. Thank you for the opportunity to review this research again. I acknowledge that the authors have made numerous revisions in response to reviewer feedback, particularly regarding the framing of the study and the analysis and interpretation of results. However, these revisions leave fundamental issues concerning the positioning and significance of this research unresolved, and raise additional concerns.

   The primary issue is that the shallow discussion in the Introduction and Discussion remains unaddressed. While the authors' decision to remove the brief revision discussion and focus on the relationship between reciprocity and intention has improved alignment between the Introduction and the experimental content, the literature review remains superficial. As Reviewers 3 and 4 noted in the previous round, prosocial behavior and reciprocity are extensively researched topics. Due to the insufficient review and discussion of this substantial body of work, it remains unclear what novel contribution this study makes to this well-established field.

   More specifically, the Introduction notes that numerous studies have already demonstrated that young children consider agents' intentions when engaging in prosocial and reciprocal behaviors. What, then, distinguishes this study from prior work? Why does the current research question arise from these existing findings? The current Introduction presents prior findings and the current study in somewhat disconnected manner, obscuring the study's significance.

**Response:** Thank you for this feedback. We have worked to improve the flow, clarity, and depth of our introduction and discussion. We agree that the study's significance was previously muddied by the organization. We have now improved the flow of both our introduction and discussion and frame the significance of our study more explicitly. The key point is perhaps expressed most succinctly on p. 6: “We therefore ask how children reason about an agent when a self-interested motivation for a seemingly prosocial action is revealed only *after* the fact. Since the prosocial action has already taken place and children will have already received its benefit, children must decide how to balance the prosocial action itself and the intention underlying the action in their reciprocity decisions.”

2. In reviewing the literature on prosociality and reciprocity, findings from infants, toddlers, and preschoolers are sometimes conflated under the term "young children." Given that age is crucial for understanding development, the review should clearly specify which developmental stage each finding pertains to.

**Response:** Thank you for pointing this out. We have added specific ages for findings in both our introduction and discussion to clarify these ages and strengthen our argument.

3. The Introduction presents research on adult behavioral economics and introduces the homo moralis model, which appears irrelevant to this study. If this model underlies the research and testing its applicability to children is important, this should be discussed in the Discussion section (currently, it is mentioned only in this Introduction paragraph). Otherwise, it is misleading and should either be removed or reframed.

**Response**: We agree that the way the homo moralis model as presented seemed disconnected at best. We have incorporated the model into the introduction as a smaller point that reinforces these ideas as connected to children. It now reads as follows:

“Reciprocity decisions and social evaluations therefore require children to balance sources of information, including benefits they may receive from others’ actions and the intentions that underlie these actions. This creates a possible tension between evaluating what an agent did versus why they did so. In adults, similar tensions have been proposed in the theoretical evolutionary model the homo moralis, which demonstrates that adults reason with a balance between self-interest and a preference for moral behavior [54-55]. Exploring whether similar reasoning exists in early childhood may provide insight into how young children consider and navigate both actions and intentions in their social decision-making.”

4. The rationale for including "willingness to play with E2" and "social bonding" as dependent variables is unclear. I understood that prize allocation to E2 measures reciprocity and liking scores assess evaluation of E2. The purpose of the remaining two measures needs explanation in either the Introduction or Methods. Clarifying this would enable deeper discussion of why reciprocity showed condition differences while the other three measures did not.

**Response**: We agree that this distinction was confusing. We included social bonding as a behavioral measure of social evaluation and thus have clarified this and collapsed social bonding into an element of social evaluation for conceptual purposes.

5. The Methods section remains unclear in places. For example, were questions about reciprocity, social evaluation, and social bonding counterbalanced? How was the new toy (colorful bouncy ball) introduced to children?

**Response**: We have now added additional information about the order of the measures, the rationale for this order, and more information about the framing of the reciprocity measure and the conclusion of the social bonding measure.

6. P-value reporting is inconsistent (e.g., p = 0.009, p < 0.01, p = .812). Please standardize the format for accurate reporting.

**Response**: Thank you for pointing this out. We inaccurately reported a *p*-value of *p* <0.01. It has now been corrected to be *p* <.001. We have corrected all other statistics to follow APA style, reporting the exact p-value unless *p* < .001 and using leading zeros only when the statistic is less than 0 but can exceed 1.

7. Lines 279-281 (p. 14) state: "Taken together, our results provide evidence that children considered the prosocial action, the revealed intention, and their own personal benefits." However, the experiment does not demonstrate consideration of personal benefits. Since the ball given to E2 belonged to E1, not the participant, it had no impact on the participant's personal benefits.

**Response**: We did not intend for this sentence to be interpreted in this way, but we now understand that it was confusing. We have reworded this to reflect that the benefit arose from the initial prosocial action. This sentence now reads as follows:

“Taken together, our results provide evidence that children considered both the benefit of the prosocial action and the later revealed intention behind the prosocial action.”

8. Lines 300-307 (p. 15) propose belief revision to explain why only reciprocity was affected by intention. However, this explanation is insufficient for why reciprocity alone was influenced. Moreover, given the partial effects observed, it is premature to conclude that children revise moral beliefs similarly to physical and social beliefs. If discussing belief revision, you must explain why intention information revised beliefs about the prosocial act but left beliefs about the agent unaffected.

**Response**: We agree. We have added justification for why we believe revision about the action but not the agent may occur and we have posited this more theoretically, as an area for future exploration.

9. In summary, while the authors' substantial revisions have improved the manuscript from its initial version, it remains unclear what contribution this research makes to the extensive existing literature on this topic.

**Response**: Thank you for your comments. We have addressed everything mentioned and believe the manuscript is tighter and stronger now. We thank you for your careful consideration.

Thank you all again for the thoughtful attention the editor and reviewers gave our manuscript over these rounds of revisions. We believe that our manuscript is theoretically and empirically stronger and sounder now thanks to these revisions.

Sincerely,

The Authors
